# Evaluation of combinatorial *cis*-regulatory elements for stable gene expression in chicken cells

**DOI:** 10.1186/1472-6750-10-69

**Published:** 2010-09-19

**Authors:** Hee W Seo, Tae M Kim, Jin W Choi, Beom K Han, Gwonhwa Song, Jae Y Han

**Affiliations:** 1 WCU Biomodulation Major, Department of Agricultural Biotechnology and Research Institute for Agriculture and Life Sciences, Seoul National University, Seoul 151-921, Korea; 2 Avicore Biotechnology Institute, Optifarm Solution Inc., Hanlim Human Tower #707, Gyeonggi-do, 435-050, Korea

## Abstract

**Background:**

Recent successes in biotechnological application of birds are based on their unique physiological traits such as unlimited manipulability onto developing embryos and simple protein constituents of the eggs. However it is not likely that target protein is produced as kinetically expected because various factors affect target gene expression. Although there have been various attempts to minimize the silencing of transgenes, a generalized study that uses multiple cis-acting elements in chicken has not been made. The aim of the present study was to analyze whether various cis-acting elements can help to sustain transgene expression in chicken fibroblasts.

**Results:**

We investigated the optimal transcriptional regulatory elements for enhancing stable transgene expression in chicken cells. We generated eight constructs that encode enhanced green fluorescent protein (eGFP) driven by either CMV or CAG promoters (including the control), containing three types of key regulatory elements: a chicken lysozyme matrix attachment region (cMAR), 5'-DNase I-hypersensitive sites 4 (cHS4), and the woodchuck hepatitis virus posttranscriptional regulatory element (WPRE). Then we transformed immortalized chicken embryonic fibroblasts with these constructs by electroporation, and after cells were expanded under G418 selection, analyzed mRNA levels and mean fluorescence intensity (MFI) by quantitative real-time PCR and flow cytometry, respectively. We found that the copy number of each construct significantly decreased as the size of the construct increased (R^2 ^= 0.701). A significant model effect was found in the expression level among various constructs in both mRNA and protein (P < 0.0001). Transcription with the CAG promoter was 1.6-fold higher than the CMV promoter (P = 0.027) and the level of eGFP expression activity in cMAR- or cHS4-flanked constructs increased by two- to three-fold compared to the control CMV or CAG promoter constructs. In addition, flow cytometry analysis showed that constructs having *cis*-acting elements decreased the level of gene silencing as well as the coefficient of variance of eGFP-expressing cells (P < 0.0001).

**Conclusions:**

Our current data show that an optimal combination of *cis*-acting elements and promoters/enhancers for sustaining gene expression in chicken cells is suggested. These results provide important information for avian transgenesis and gene function studies in poultry.

## Background

The delivery of gene constructs into animal cells is an indispensible tool for conducting various biomedical studies and producing transgenic animals. However, several aspects should be taken into consideration for successful transgene expression in target cells. The extent of transgene expression largely depends on multiple aspects, such as gene delivery method, cellular physicochemical properties, and the traits of the construct [1]. Although methods for gene transfer into the host cells are currently standarized in several cell types, the transfection efficiency remains unsatisfactory in many cases and efforts to devise an optimal construct that can induce constant expression have not been very promising. In addition, many obstacles, such as transgene silencing and variegation, have yet to be overcome to enhance transgene expression [2].

To date, various strong enhancers/promoters have been used for stable expression of transgenes in animal cells. Among these, the cytomegalovirus (CMV) immediate-early enhancer/promoter and CAG (CMV enhancer with a chicken beta-actin transcription start site and a rabbit beta-globin intron) promoters have been used in a variety of cells due to their ability to induce immediate and strong transcription [3,4]. However, the two promoters exhibit different transcriptional activities, presumably due to distinctive constituents [5-7]. Other studies have also shown transcriptional variation among different tissues or developmental stages [8-11].

Other transcription regulator elements have also been used to sustain transcription activity. Chicken 5'-DNase I-hypersensitive sites 4 (cHS4), derived from the chicken beta-globin locus, contains GC-rich DNA sequences and a CTCF-dependent element linked to the nuclear matrix [12,13]. It enhances transgene expression in cultured cells [14] and transgenic animal cells [15], and prevents silencing of viral vectors [16,17]. The woodchuck hepatitis virus posttranscriptional regulatory element (WPRE) is derived from the 3' untranslated region (3' UTR) of viral RNA [18] and acts as a posttranscriptional enhancer by stimulating the cytoplasmic import of mRNAs [19,20]. The matrix attachment region (cMAR) from the 5' regulatory region of the chicken lysozyme gene contains AT-rich sequences and enhances transgene expression in various immortalized cells [21,22], transgenic animals [23,24], and plants [25].

Birds serve as excellent models of disease and bioreactor production due to the ease of embryo manipulation and the availability of various transgenic technologies using primordial germ cells and testicular cells [26]. However, only a limited number of recently refined constructs carrying transcription activators have been used for inducing stable gene expression in transgenic birds. Therefore, we evaluated whether these multiple transcription regulators can help maintain stable gene expression in chicken cells, and propose an optimal construct with an optimal combination of promoter/enhancer and transcription regulatory units.

## Results

### Correlation between size and copy number of the delivered plasmids

**Figure 1:**
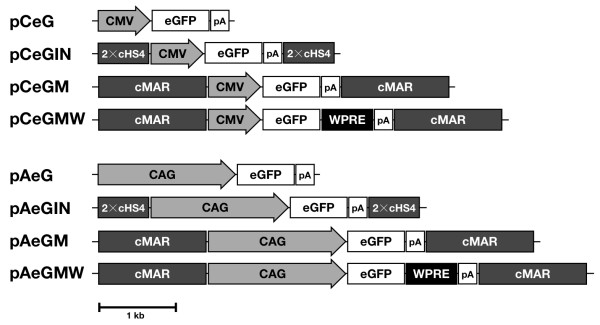
**Construction of the vectors containing different *cis*-regulatory elements**. CMV, human cytomegalovirus immediate-early enhancer/promoter; CAG, CMV enhancer + chicken beta-actin promoter + rabbit beta-globin intron; eGFP, enhanced green fluorescent protein; pA, bovine growth hormone polyadenylation signal; cMAR, chicken lysozyme matrix attachment region (BamHI-PvuI fragment); cHS4, the core region of chicken beta-globin insulator (250bp); WPRE, woodchuck hepatitis virus posttranscriptional regulatory element.

**Figure 2:**
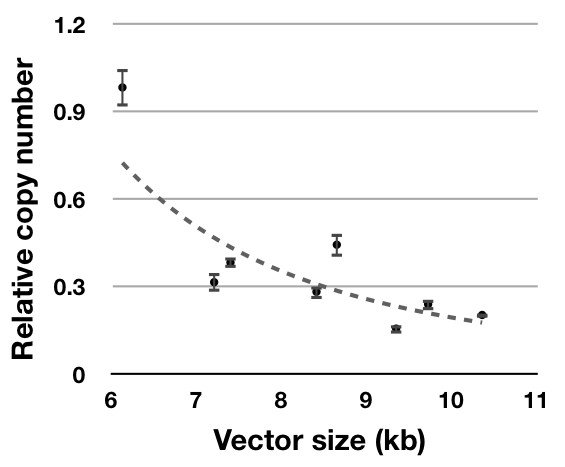
**Correlation between relative copy number and vector size**. Relative vector copy number, analyzed by real-time PCR, decreased exponentially as vector size increased. The graph and R^2 ^were created with Microsoft Office Excel.

Given that each vector contained different promoters and *cis*-acting elements, their sizes were variable (Fig. 1). Therefore, we examined the relationship between vector size and the integrated vector copy numbers. Relative vector copy numbers were generally reduced as vector size increased (R^2 ^= 0.701; Fig. 2).

### CMV and CAG promoter transcription activities differ in chicken embryonic fibroblasts

**Figure 3:**
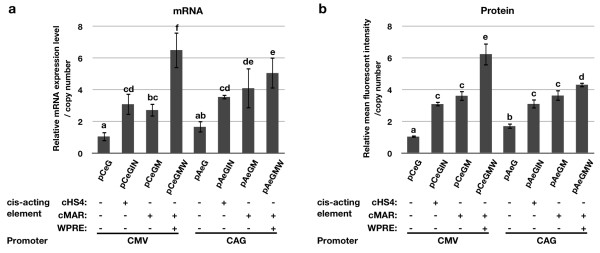
**Analysis of eGFP expression driven by CMV or CAG promoter in DF-1 cells**. The effect of various *cis*-acting elements on eGFP expression were analyzed in both mRNA (a) and protein (b) levels. A significant model effect was found on both mRNA and MFI (both P < 0.0001). Means with different superscripts (a, b, c, d, e, f) differ significantly (P < 0.05; F values = 15.80 and 67.51 in mRNA and protein level analysis, respectively; total degree of freedom = 23). Data represent the mean of three replicates, and error bars indicate ± S.D. (*n *= 3 in each plasmid).

To compare the strength of the CMV and CAG promoters, mRNA levels were measured by real-time PCR. MFI was analyzed by flow cytometry to study transgene expression levels induced by the CMV and CAG promoters in DF-1 cells. The difference in mRNA expression levels between the CMV and CAG promoters was not significant (P = 0.35), whereas the MFI of the CAG promoter (pAeG) was 1.6-fold higher than that of the CMV promoter (pCeG) (P = 0.027; Fig. 3a, b). When HS4 was juxtaposed to the 5' and 3' regions of the transgene cassette, the difference in mRNA levels and MFI values between the two promoters was not significant (P > 0.05; Fig. 3a, b). However, the transcript level generated by the CAG promoter was 1.5-fold higher than that generated by CMV in MAR-flanked plasmids (pCeGM versus pAeGM, P = 0.0439; Fig. 3a).

### cMAR and cHS4 enhance transgene expression

**Figure 4:**
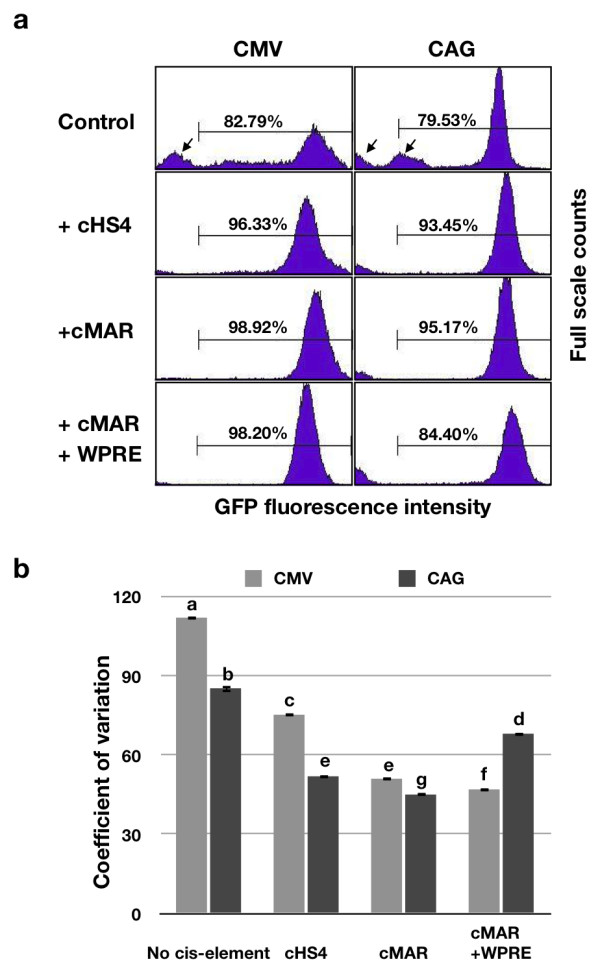
**Flow cytometry analysis of electroporated DF-1 cells**. (a) The proportion of eGFP-expressing cells are indicated. Arrows denote cells of the non-expressing population. (b) The effect of *cis*-acting elements on the coefficient of variation (CV) in eGFP expression driven by CMV and CAG promoters. A significant model effect was found on CV among the eight constructs (P < 0.0001). Means with different letters (a, b, c, d, e, f, g) differ significantly (P < 0.05; F value = 2927.53; total degree of freedom = 23). Data represent the mean of three replicates, and error bars indicate ± S.D.(*n *= 3 in each plasmid).

Both cMAR and cHS4 increased eGFP expression in mRNA and protein (Fig. 3a, b). The CMV promoter led to increases of 2.9-fold and more than 3.0-fold in mRNA (P = 0.0052) and MFI (P < 0.0001), respectively, in pCeGIN compared to those of the control pCeG. Adding cMAR (pCeGM) yielded an approximately 2.6-fold increase in mRNA expression level (P = 0.018) and 3.5-fold increase in MFI value (P < 0.0001) relative to pCeG. The CAG promoter led to increases of 2.1-fold (P = 0.0086) and 1.9-fold (P = 0.0001), respectively, in the HS4-flanked plasmid (pAeGIN) compared to the control pAeG. Likewise, cMAR led to increases of 2.5-fold (P = 0.0014) and 2.1-fold (P < 0.0001), respectively, in pAeGM compared to pAeG. To investigate the effects of cHS4 and cMAR on eGFP expression at the single-cell level, flow cytometry was performed. A considerable proportion of the cells with transgene suppression were noted in control plasmids (Fig. 4a, arrows); however, adding cHS4 or cMAR clearly reduced the level of such heterogeneity. Furthermore, the coefficient of variation (CV) of eGFP-expressing cells was reduced in the vector with cHS4 and cMAR (P < 1 × 10^-5^; Fig. 4b).

### The effect of WPRE is promoter-dependent

pCeGMW increased eGFP expression in both mRNA (2.4-fold, P < 0.0001) and MFI (1.7-fold, P < 0.0001) compared to the control pCeGM (Fig. 3a, b). With the CAG promoter, pAeGMW did not induce a dramatic increase in mRNA level (P = 0.1482), whereas an increase was noted in the MFI compared to pAeGM (1.2 folds, P = 0.0238). The level of transcription silencing in each construct was then analyzed by flow cytometry, which revealed an increase in the proportion of eGFP-positive cells in HS4-, MAR-, and MAR+WPRE-inserted constructs compared to those transfected with control plasmids (pCeG and pAeG). However, in pAeGMW, the eGFP-positive population decreased compared to those of pAeGM and pAeGIN. A significant model effect was found on the CV of eight constructs that were tested (P < 0.0001). Generally, the CAG promoter constituted less heterogeneity in eGFP expression compared to CMV, except in the construct with MAR and WPRE, for which the CV of CAG (pAeGMW) was higher than that of CMV (pCeGMW, Fig. 4b).

## Discussion

Birds are an excellent model for studying transgenic animals and disease because of their physiological uniqueness during embryogenesis [26-29]. Various methods for producing transgenic birds have been developed over the last two decades [30-33] however, optimized gene constructs are lacking. Therefore, to develop an efficient non-viral vector system in chicken, we examined three regulatory elements for high transgene expression: (1) promoter/enhancer elements, (2) *cis*-acting elements for blocking the position effect, and (3) posttranscriptional elements associated with RNA processing.

We described and compared the role of the various *cis*-acting elements as well as the efficacy of combinatorial regulatory elements to allow for enhanced regulation of transfected genes. To identify optimal combinations of *cis*-acting elements for efficient transgene expression, we constructed eight non-viral vectors containing various *cis*-regulatory elements. Furthermore, we compared the CMV and CAG promoters to identify the most appropriate promoter for enhancing transgene expression. Considering the position effects caused by the chromosomal environment in the host cells, cMAR and cHS4 derived from chicken genomic sequences were tested. In addition, WPRE was inserted into the downstream region of the open reading frame of the eGFP gene to increase transgene expression levels via post-transcriptional regulation of transcripts.

The relative vector copy numbers decreased as vector size increased, which has also been reported in previous studies. For example, the gene-transfer capacity of plasmid DNA has been shown to be inversely related to the vector size, and furthermore, gene expression decreases as the size of DNA increases [34,35]. Given that the electroporation performed in the present study contributed to the delivery of plasmid DNA into the cytoplasm, our results may be due to a decrease in DNA diffusion mobility from the cytoplasm to the nucleus as the vector size increased [36,37].

The CAG promoter was more effective than the CMV promoter for transgene expression in DF-1 cells, possibly due to differences in the promoter constituents. The CMV promoter originated from the immediate-early gene of human CMV did not contain the intron sequence [38], whereas the CAG promoter consisted of the same CMV immediate-early enhancer with a chicken beta-actin promoter and rabbit beta-globin intron [5]. The presence of an intron in the promoter affects transgene expression in mammalian cells [5], and inserting a large CMV intron and beta-actin intron into plasmids with the same promoter increases luciferase activity [6]. Therefore, our results support previous studies showing the roles of the chicken beta-actin promoter and rabbit beta-globin introns in the CAG promoter in improving the efficiency of transgene expression [5].

The chicken HS4 insulator, which contains a CTCF-binding site, enters the nuclear matrix and forms nucleosome gaps by binding with CTCF [39]. The expression of the transgene cassette flanked by the HS4 insulator is not affected by other neighboring chromosomal elements such as enhancers [40]. The MAR element, which consists of AT-rich DNA, makes an independent loop or domain in the chromatin by binding to nuclear matrix proteins such as topoisomerase II, histone H1, lamins, SP120, ARBP, and SATB1. Using this mechanism, interferences with chromosomal structures surrounding the gene cassettes are blocked, thereby enhancing the binding of the transcription factor to the regulatory region [41]. In the present study, we also found that cHS4 and cMAR increased the number of transfected cells and reduced variation in eGFP expression. Therefore, our data indicate that 2× cHS4 and cMAR prevent position effects that exert an inhibitory effect on transgene expression in chicken cells.

WPRE, which is associated with RNA processing, has a pivotal regulatory element for efficient transgene expression. It can enhance transgene expression related to splicing and polyadenylation, export it into the cytoplasm, and stabilize the mRNA transcript [18]. When combined with the appropriate regulatory elements, WPRE promotes the cytoplasmic export of nuclear mRNA without introns [42,43]. In addition, when inserted into the 3' UTR of non-viral vectors, it increases transgene expression in mammalian cells [44]. Our current results showed that WPRE improves eGFP expression driven by CMV and CAG promoters in DF-1 cells. However, this effect is promoter-dependent. When WPRE was added, the increase in eGFP expression was higher in the vector combined with the CMV promoter than with CAG. These results support the idea that the effect of WPRE depends on what constitutes the promoter [45].

We have produced germline chimeric chicken [46-49] and quail [50,51] using various types of germ cells to establish avian transgenesis. Among various donor cells that generate chimeras, the potential of primordial germ cells (PGCs) is limitless based on their ability to migrate towards the sex cord in the recipient embryo, and subsequent testcross analyses allows the production of donor-derived offspring. However, there are several obstacles to this process because germ cells are relatively transcriptionally quiescent and prone to switching off transgene expression [52,53]. Our results can be directly applied to genetic and cellular manipulation systems for transgenesis via an increase in transcription activity, thus preventing position effects provoked by nearby enhancers/suppressors and increasing cytoplasmic export of transcripts by stabilizing mRNA.

## Conclusions

The purpose of this research was to develop a non-viral vector system with minimized transgene silencing in chicken cells. The application of cHS4 and cMAR effectively reduced the silencing of transgene expression, while WPRE enhanced the expression level. Our data constitute optimized regulatory elements that can be used to induce stable gene expression and study gene function in chicken. The present research also provides new insights to establish the bioreactor and model bird production system using avian transgenic technology.

## Methods

### Vector construction

To construct pCeG, the *EcoR *I-*Xba *I fragment of eGFP from pEGFP-1 (Clontech, Mountain View, CA) was inserted into pcDNA3 (Invitrogen, Carlsbad, CA) containing the CMV promoter (Fig. 1). To construct pAeG, the *Spe *I-*EcoR *I fragment of the CAG promoter from pCAGGS [5] was inserted into pCeG. The BP-MAR fragment [54] was derived from the PCR product of chicken genomic DNA using the primer set P1 (5'-CGC TCT AGA ACT AGT GGG ATC CAT-3') and P2 (5'-ATG CCT GTT GCA GCT GTT TAC G-3'), which was then inserted into *Bgl *II and *Dra *III sites of pCeG and pAeG, thus constructing pCeGM and pAeGM, respectively. pCeGIN and pAeGIN were constructed inserting the tandem-duplicated cHS4 (2×HS4) fragment, which was amplified from chicken genomic DNA using P3 (5'-GCA GGT TTC CTG GAA GGT-3') and P4 (5'-AGC TAA AGC TTT TTC CGT-3') primers, into *Bgl *II and *Dra *III sites of pCeG and pAeG, respectively [55]. To construct pCeGMW and pAeGMW, the WPRE fragment was amplified from pWPI by PCR using P5 (5'-GCG GCC GCG ACC TCG AGG GAA TTC CGA T-3') and P6 (5'-GCG GCC GCT TCG AAG CTT GAC GAA TTC C-3') primers. The PCR product was then inserted into a *Not *I site of pCeGM and pAeGM. A schematic diagram of the vectors is illustrated in Figure 1.

### Cell culture and transfection

DF-1 (ATCC, Manassas, VA, Cat. No. CRL-12203) cells were cultured in Dulbecco's Modified Eagle medium (Invitrogen, Carlsbad, CA) supplemented with 10% fetal bovine serum (FBS, Invitrogen) and 1× antibiotic-antimycotics (Invitrogen) in an incubator at 37°C and 5% CO_2 _in an air atmosphere with 60-70% relative humidity. Transfection was performed as described previously [56]. Briefly, a total of 1×10^6 ^of cells were suspended in serum-free OPTIMEM-I (Invitrogen) with the same molar ratio of linearized plasmid DNAs in a 0.4-cm cuvette (Bio-Rad, Hercules, CA) and electroporation was performed using Gene Pulser Xcell™ (Bio-Rad). The transfected cells were selected with 400 μg/ml G418 (Invitrogen) for 21 days and cultured at least 90 days before further analysis. Three cell lines in each plasmid were subjected to analysis of eGFP expression.

### Quantitative real-time PCR

Total RNA was isolated from G418-selected cells using TRIZOL^® ^Reagent (Invitrogen). cDNA was synthesized using the SuperScript^® ^III First-Strand Synthesis kit (Invitrogen) according to the manufacturer's protocol. Genomic DNA was extracted from cells using Wizard^® ^SV Genomic DNA purification system (Promega, Madison, WI) according to the manufacturer's instructions. The expression level of mRNA from the cells electroporated with various constructs were compared to those with pCeG and measured in triplicate using real-time PCR (ABI 7300 Real-Time PCR system, Applied Biosystems, Foster City, CA) and SYBR^® ^Green (Sigma, St. Louis, MO). To measure and compare relative mRNA expression levels, the measured values were divided by the copy numbers of their relative vectors [45,54]. The primers used for quantitative PCR were eGFP-forward (5'-TCA AGG ACG ACG GCA ACT ACA A-3'), eGFP-reverse (5'-GAT GGG GGT GTT CTG CTG GT-3'), GAPDH-forward (5'-TCA CAG CCA CAC AGA AGA CGG-3'), and GAPDH-reverse (5'-CAG ACG GCA GGT CAG GTC AA-3'). Relative quantification of mRNA expression was calculated using the 2^-ΔΔCt ^method [57].

### Flow cytometry

Flow cytometry was performed using a FACS Calibur™ flow cytometer (BD Biosciences, San Jose, CA) and CellQuest software (BD Biosciences) equipped with a standard fluorescence filter set. Trypsinized cells were resuspended in phosphate buffered saline (PBS, Invitrogen) supplemented with 3% FBS. The mean fluorescence intensity (MFI) and coefficient of variation (CV) were also obtained by flow cytometry.

### Statistical analysis

Data were subjected to analysis of variance (ANOVA) according to the general linear model (PROC-GLM) of the SAS program (SAS Institute, Cary, NC). If the main effect was significant, treatment effects were compared by the least squares method. P-values of less than 0.05 were considered statistically significant.

## Authors' contributions

JYH, as a Corresponding author, conceived of the study, and participated in its design, collected data, interpreted the research results and edited the manuscript. JYH supervised HWS, prepared and submitted the manuscript. HWS designed and constructed plasmids, carried out the chicken cell culture and transfection, performed quantitative real-time PCR and flow cytomestry, and conducted the statistical analysis of data. HWS drafted and edited the manuscript. TMK participated with the statistical analysis and interpretation of data, provided the advice on plasmid design and transfection of chicken cells, and edited the manuscript. JWC provided technical assistance with quantitative real-time PCR, participated with manuscript preparation. BKH provided the advice on background studies, data interpretation and manuscript editing. GS provided the advice on interpretation of data, corrected and edited the manuscript. The final manuscript was read and approved by all authors.

## References

[B1] Ausubel FM Short protocols in molecular biology: a compendium of methods from Current protocols in molecular biology 1999 4 New York: Wiley

[B2] Ellis J Silencing and variegation of gammaretrovirus and lentivirus vectors Hum Gene Ther 2005 16 11 1241 1246 10.1089/hum.2005.16.1241 16259557

[B3] Ward CM Stern PL The human cytomegalovirus immediate-early promoter is transcriptionally active in undifferentiated mouse embryonic stem cells Stem Cells 2002 20 5 472 475 10.1634/stemcells.20-5-472 12351818

[B4] Nitta Y Kawamoto S Halbert C Iwata A Miller AD Miyazaki J Allen MD A CMV-actin-globin hybrid promoter improves adeno-associated viral vector gene expression in the arterial wall in vivo J Gene Med 2005 7 10 1348 1355 10.1002/jgm.784 15945122

[B5] Niwa H Yamamura K Miyazaki J Efficient selection for high-expression transfectants with a novel eukaryotic vector Gene 1991 108 2 193 199 10.1016/0378-1119(91)90434-D 1660837

[B6] Xu ZL Mizuguchi H Ishii-Watabe A Uchida E Mayumi T Hayakawa T Optimization of transcriptional regulatory elements for constructing plasmid vectors Gene 2001 272 1-2 149 156 10.1016/S0378-1119(01)00550-9 11470520

[B7] Wang S Hu C Zhu J Transcriptional silencing of a novel hTERT reporter locus during in vitro differentiation of mouse embryonic stem cells Mol Biol Cell 2007 18 2 669 677 10.1091/mbc.E06-09-0840 17151355 PMC1783791

[B8] Ramezani A Hawley TS Hawley RG Lentiviral vectors for enhanced gene expression in human hematopoietic cells Mol Ther 2000 2 5 458 469 10.1006/mthe.2000.0190 11082319

[B9] Hong S Hwang DY Yoon S Isacson O Ramezani A Hawley RG Kim KS Functional analysis of various promoters in lentiviral vectors at different stages of in vitro differentiation of mouse embryonic stem cells Mol Ther 2007 15 9 1630 1639 10.1038/sj.mt.6300251 17609656 PMC2614215

[B10] Liu Y Okada T Nomoto T Ke X Kume A Ozawa K Xiao S Promoter effects of adeno-associated viral vector for transgene expression in the cochlea in vivo Exp Mol Med 2007 39 2 170 175 17464178 10.1038/emm.2007.19

[B11] Xia X Zhang Y Zieth CR Zhang SC Transgenes delivered by lentiviral vector are suppressed in human embryonic stem cells in a promoter-dependent manner Stem Cells Dev 2007 16 1 167 176 10.1089/scd. 2006.0057 17348812 10.1089/scd.2006.0057 PMC2801347

[B12] Walters MC Fiering S Bouhassira EE Scalzo D Goeke S Magis W Garrick D Whitelaw E Martin DI The chicken beta-globin 5'HS4 boundary element blocks enhancer-mediated suppression of silencing Mol Cell Biol 1999 19 5 3714 3726 10207095 10.1128/mcb.19.5.3714 PMC84188

[B13] Burgess-Beusse B Farrell C Gaszner M Litt M Mutskov V Recillas-Targa F Simpson M West A Felsenfeld G The insulation of genes from external enhancers and silencing chromatin Proc Natl Acad Sci USA 2002 99 Suppl 4 16433 16437 10.1073/pnas.162342499 12154228 PMC139905

[B14] Villemure JF Savard N Belmaaza A Promoter suppression in cultured mammalian cells can be blocked by the chicken beta-globin chromatin insulator 5'HS4 and matrix/scaffold attachment regions J Mol Biol 2001 312 5 963 974 10.1006/jmbi.2001.5015 11580242

[B15] Rival-Gervier S Pantano T Viglietta C Maeder C Prince S Attal J Jolivet G Houdebine LM The insulator effect of the 5'HS4 region from the beta-globin chicken locus on the rabbit WAP gene promoter activity in transgenic mice Transgenic Res 2003 12 6 723 730 10.1023/B:TRAG.0000005242.72076.d1 14713201

[B16] Jakobsson J Rosenqvist N Thompson L Barraud P Lundberg C Dynamics of transgene expression in a neural stem cell line transduced with lentiviral vectors incorporating the cHS4 insulator Exp Cell Res 2004 298 2 611 623 10.1016/j.yexcr.2004.04.037 15265707

[B17] Aker M Tubb J Groth AC Bukovsky AA Bell AC Felsenfeld G Kiem HP Stamatoyannopoulos G Emery DW Extended core sequences from the cHS4 insulator are necessary for protecting retroviral vectors from silencing position effects Hum Gene Ther 2007 18 4 333 343 10.1089/hum.2007.021 17411365

[B18] Donello JE Loeb JE Hope TJ Woodchuck hepatitis virus contains a tripartite posttranscriptional regulatory element J Virol 1998 72 6 5085 5092 9573279 10.1128/jvi.72.6.5085-5092.1998 PMC110072

[B19] Loeb JE Cordier WS Harris ME Weitzman MD Hope TJ Enhanced expression of transgenes from adeno-associated virus vectors with the woodchuck hepatitis virus posttranscriptional regulatory element: implications for gene therapy Hum Gene Ther 1999 10 14 2295 2305 10.1089/10430349950016942 10515449

[B20] Mastroyiannopoulos NP Feldman ML Uney JB Mahadevan MS Phylactou LA Woodchuck post-transcriptional element induces nuclear export of myotonic dystrophy 3' untranslated region transcripts EMBO Rep 2005 6 5 458 463 10.1038/sj.embor.7400390 15832171 PMC1299300

[B21] Phi-Van L von Kries JP Ostertag W Stratling WH The chicken lysozyme 5' matrix attachment region increases transcription from a heterologous promoter in heterologous cells and dampens position effects on the expression of transfected genes Mol Cell Biol 1990 10 5 2302 2307 2325653 10.1128/mcb.10.5.2302-2307.1990 PMC360577

[B22] Girod PA Zahn-Zabal M Mermod N Use of the chicken lysozyme 5' matrix attachment region to generate high producer CHO cell lines Biotechnol Bioeng 2005 91 1 1 11 10.1002/bit.20563 15889435

[B23] McKnight RA Shamay A Sankaran L Wall RJ Hennighausen L Matrix-attachment regions can impart position-independent regulation of a tissue-specific gene in transgenic mice Proc Natl Acad Sci USA 1992 89 15 6943 6947 10.1073/pnas.89.15.6943 1495984 PMC49621

[B24] Castilla J Pintado B Sola I Sanchez-Morgado JM Enjuanes L Engineering passive immunity in transgenic mice secreting virus-neutralizing antibodies in milk Nat Biotechnol 1998 16 4 349 354 10.1038/nbt0498-349 9555725 PMC7097410

[B25] Oh SJ Jeong JS Kim EH Yi NR Yi SI Jang IC Kim YS Suh SC Nahm BH Kim JK Matrix attachment region from the chicken lysozyme locus reduces variability in transgene expression and confers copy number-dependence in transgenic rice plants Plant Cell Rep 2005 24 3 145 154 10.1007/s00299-005-0915-2 15714322

[B26] Han JY Germ cells and transgenesis in chickens Comp Immunol Microbiol Infect Dis 2009 32 2 61 80 10.1016/j.cimid.2007.11.010 18249442

[B27] Ivarie R Competitive bioreactor hens on the horizon Trends Biotechnol 2006 24 3 99 101 10.1016/j.tibtech.2006.01.004 16445998

[B28] Smith CA Roeszler KN Ohnesorg T Cummins DM Farlie PG Doran TJ Sinclair AH The avian Z-linked gene DMRT1 is required for male sex determination in the chicken Nature 2009 461 7261 267 271 10.1038/nature08298 19710650

[B29] Zhao D McBride D Nandi S McQueen HA McGrew MJ Hocking PM Lewis PD Sang HM Clinton M Somatic sex identity is cell autonomous in the chicken Nature 2010 464 7286 237 U115 10.1038/nature08852 20220842 PMC3925877

[B30] Chapman SC Lawson A Macarthur WC Wiese RJ Loechel RH Burgos-Trinidad M Wakefield JK Ramabhadran R Mauch TJ Schoenwolf GC Ubiquitous GFP expression in transgenic chickens using a lentiviral vector Development 2005 132 5 935 940 10.1242/dev.01652 15673573

[B31] Koo BC Kwon MS Choi BR Kim JH Cho SK Sohn SH Cho EJ Lee HT Chang W Jeon I Park JK Park JB Kim T Production of germline transgenic chickens expressing enhanced green fluorescent protein using a MoMLV-based retrovirus vector FASEB J 2006 20 13 2251 2260 10.1096/fj.06-5866com 17077302

[B32] van de Lavoir MC Diamond JH Leighton PA Mather-Love C Heyer BS Bradshaw R Kerchner A Hooi LT Gessaro TM Swanberg SE Delany ME Etches RJ Germline transmission of genetically modified primordial germ cells Nature 2006 441 7094 766 769 10.1038/nature04831 16760981

[B33] Shin SS Kim TM Kim SY Kim TW Seo HW Lee SK Kwon SC Lee GS Kim H Lim JM Han JY Generation of transgenic quail through germ cell-mediated germline transmission FASEB J 2008 22 7 2435 2444 10.1096/fj.07-101485 18263695

[B34] Kreiss P Cameron B Rangara R Mailhe P Aguerre-Charriol O Airiau M Scherman D Crouzet J Pitard B Plasmid DNA size does not affect the physicochemical properties of lipoplexes but modulates gene transfer efficiency Nucleic Acids Res 1999 27 19 3792 3798 10.1093/nar/27.19.3792 10481017 PMC148641

[B35] Yin W Xiang P Li Q Investigations of the effect of DNA size in transient transfection assay using dual luciferase system Anal Biochem 2005 346 2 289 294 10.1016/j.ab.2005.08.029 16213455

[B36] Lukacs GL Haggie P Seksek O Lechardeur D Freedman N Verkman AS Size-dependent DNA mobility in cytoplasm and nucleus J Biol Chem 2000 275 3 1625 1629 10.1074/jbc.275.3.1625 10636854

[B37] Lechardeur D Verkman AS Lukacs GL Intracellular routing of plasmid DNA during non-viral gene transfer Adv Drug Deliv Rev 2005 57 5 755 767 10.1016/j.addr.2004.12.008 15757759

[B38] Hennighausen L Fleckenstein B Nuclear factor 1 interacts with five DNA elements in the promoter region of the human cytomegalovirus major immediate early gene EMBO J 1986 5 6 1367 1371 3015602 10.1002/j.1460-2075.1986.tb04368.x PMC1166949

[B39] Yusufzai TM Felsenfeld G The 5'-HS4 chicken beta-globin insulator is a CTCF-dependent nuclear matrix-associated element Proc Natl Acad Sci USA 2004 101 23 8620 8624 10.1073/pnas.0402938101 15169959 PMC423244

[B40] Zhao H Kim A Song SH Dean A Enhancer blocking by chicken beta-globin 5'-HS4: role of enhancer strength and insulator nucleosome depletion J Biol Chem 2006 281 41 30573 30580 10.1074/jbc.M606803200 16877759

[B41] Boulikas T Chromatin domains and prediction of MAR sequences Int Rev Cytol 1995 162A 279 388 8575883 10.1016/s0074-7696(08)61234-6

[B42] Zufferey R Donello JE Trono D Hope TJ Woodchuck hepatitis virus posttranscriptional regulatory element enhances expression of transgenes delivered by retroviral vectors J Virol 1999 73 4 2886 2892 10074136 10.1128/jvi.73.4.2886-2892.1999 PMC104046

[B43] Gruh I Wunderlich S Winkler M Schwanke K Heinke J Blomer U Ruhparwar A Rohde B Li RK Haverich A Martin U Human CMV immediate-early enhancer: a useful tool to enhance cell-type-specific expression from lentiviral vectors J Gene Med 2008 10 1 21 32 10.1002/jgm.1122 18022932

[B44] Johansen J Tornoe J Moller A Johansen TE Increased in vitro and in vivo transgene expression levels mediated through cis-acting elements J Gene Med 2003 5 12 1080 1089 10.1002/jgm.444 14661183

[B45] Klein R Ruttkowski B Knapp E Salmons B Gunzburg WH Hohenadl C WPRE-mediated enhancement of gene expression is promoter and cell line specific Gene 2006 372 153 161 10.1016/j.gene.2005.12.018 16488559

[B46] Chang IK Jeong DK Hong YH Park TS Moon YK Ohno T Han JY Production of germline chimeric chickens by transfer of cultured primordial germ cells Cell Biol Int 1997 21 8 495 499 10.1006/cbir.1997.0173 9451806

[B47] Park TS Jeong DK Kim JN Song GH Hong YH Lim JM Han JY Improved germline transmission in chicken chimeras produced by transplantation of gonadal primordial germ cells into recipient embryos Biol Reprod 2003 68 5 1657 1662 10.1095/biolreprod.102.006825 12606438

[B48] Park TS Hong YH Kwon SC Lim JM Han JY Birth of germline chimeras by transfer of chicken embryonic germ (EG) cells into recipient embryos Mol Reprod Dev 2003 65 4 389 395 10.1002/mrd.10304 12840812

[B49] Lee YM Jung JG Kim JN Park TS Kim TM Shin SS Kang DK Lim JM Han JY A testis-mediated germline chimera production based on transfer of chicken testicular cells directly into heterologous testes Biol Reprod 2006 75 3 380 386 10.1095/biolreprod.106.052084 16723507

[B50] Kim MA Park TS Kim JN Park HJ Lee YM Ono T Lim JM Han JY Production of quail (Coturnix japonica) germline chimeras by transfer of gonadal primordial germ cells into recipient embryos Theriogenology 2005 63 3 774 782 10.1016/j.theriogenology.2004.04.014 15629796

[B51] Park TS Kim MA Lim JM Han JY Production of quail (Coturnix japonica) germline chimeras derived from in vitro-cultured gonadal primordial germ cells Mol Reprod Dev 2008 75 2 274 281 10.1002/mrd.20821 17874456

[B52] Leatherman JL Jongens TA Transcriptional silencing and translational control: key features of early germline development Bioessays 2003 25 4 326 335 10.1002/bies.10247 12655640

[B53] Seydoux G Braun RE Pathway to totipotency: lessons from germ cells Cell 2006 127 5 891 904 10.1016/j.cell.2006.11.016 17129777

[B54] Phi-Van L Stratling WH Dissection of the ability of the chicken lysozyme gene 5' matrix attachment region to stimulate transgene expression and to dampen position effects Biochemistry 1996 35 33 10735 10742 10.1021/bi9603783 8718863

[B55] Recillas-Targa F Pikaart MJ Burgess-Beusse B Bell AC Litt MD West AG Gaszner M Felsenfeld G Position-effect protection and enhancer blocking by the chicken beta-globin insulator are separable activities Proc Natl Acad Sci USA 2002 99 10 6883 6888 10.1073/pnas.102179399 12011446 PMC124498

[B56] Hong YH Moon YK Jeong DK Han JY Improved transfection efficiency of chicken gonadal primordial germ cells for the production of transgenic poultry Transgenic Res 1998 7 4 247 252 10.1023/A:1008861826681 9859213

[B57] Livak KJ Schmittgen TD Analysis of relative gene expression data using real-time quantitative PCR and the 2(T)(-Delta Delta C) method Methods 2001 25 4 402 408 10.1006/meth.2001.1262 11846609

